# Impact of Early Childhood Malnutrition on Adult Brain Function: An Evoked-Related Potentials Study

**DOI:** 10.3389/fnhum.2022.884251

**Published:** 2022-07-01

**Authors:** Kassandra Roger, Phetsamone Vannasing, Julie Tremblay, Maria L. Bringas Vega, Cyralene P. Bryce, Arielle G. Rabinowitz, Pedro A. Valdés-Sosa, Janina R. Galler, Anne Gallagher

**Affiliations:** ^1^LION Lab, Sainte-Justine University Hospital Research Center, University of Montreal, Montreal, QC, Canada; ^2^MOE Key Lab for Neuroinformation, The Clinical Hospital of Chengdu Brain Science Institute, University of Electronic Science and Technology of China, Chengdu, China; ^3^Barbados Nutrition Study, Bridgetown, Barbados; ^4^Department of Neurology and Neurosurgery, McGill University, Montreal, QC, Canada; ^5^Division of Pediatric Gastroenterology and Nutrition, MassGeneral Hospital for Children, Boston, MA, United States

**Keywords:** EEG, Go-No-Go, ERP, inhibition, attention, protein-energy malnutrition (PEM)

## Abstract

More than 200 million children under the age of 5 years are affected by malnutrition worldwide according to the World Health Organization. The Barbados Nutrition Study (BNS) is a 55-year longitudinal study on a Barbadian cohort with histories of moderate to severe protein-energy malnutrition (PEM) limited to the first year of life and a healthy comparison group. Using quantitative electroencephalography (EEG), differences in brain function during *childhood* (lower alpha1 activity and higher theta, alpha2 and beta activity) have previously been highlighted between participants who suffered from early PEM and controls. In order to determine whether similar differences persisted into *adulthood*, our current study used recordings obtained during a Go-No-Go task in a subsample of the original BNS cohort [population size (N) = 53] at ages 45–51 years. We found that previously malnourished adults [sample size (*n*) = 24] had a higher rate of omission errors on the task relative to controls (*n* = 29). Evoked-Related Potentials (ERP) were significantly different in participants with histories of early PEM, who presented with lower N2 amplitudes. These findings are typically associated with impaired conflict monitoring and/or attention deficits and may therefore be linked to the attentional and executive function deficits that have been previously reported in this cohort in childhood and again in middle adulthood.

## Introduction

According to the World Health Organization, 45% of deaths among children under 5 years of age are caused by malnutrition, which impacts more than 224 million children globally and makes this condition a critical global health concern (UNICEF et al., 2021). The situation will likely be exacerbated by the COVID-19 pandemic according to UNICEF (2021) due to deteriorations in household wealth and disruptions to the availability and affordability of nutritious food and essential nutrition services. Protein-energy malnutrition (PEM) or severe acute malnutrition (SAM) is a specific type of malnutrition defined as an acute caloric deficit due to deficiency of all macronutrients, and micronutrients in some cases (see Galler et al., 2021 for a review of the definitions).

While malnutrition has deleterious effects on cognitive abilities (Champakam et al., 1968; Galler et al., 1983b,1984a; Upadhyay et al., 1989a; Mendez and Adair, 1999; Berkman et al., 2002), it has also been associated with various effects on motor skills, social abilities and mental health (Galler et al., 1983a,1984b,^1985^; Upadhyay et al., 1989b), with many of these effects persisting into adolescence and even adulthood (Hoorweg and Stanfield, 1976; Walker et al., 2007; Galler et al., 2012b,c; Waber et al., 2014a,b). Using electroencephalography (EEG), brain function alterations have also been linked to early childhood malnutrition (for a review see Gladstone et al., 2014). Although these findings were replicated in several studies (Stoch et al., 1963; Baraitser and Evans, 1969; Griesel et al., 1990), few of them assessed the children at later timepoints in childhood. Bartel et al. (1979) studied 6- to 12-year-old children hospitalized for the treatment of kwashiorkor during the first 27 months of life and confirmed that their rest EEGs still exhibited significantly diminished alpha and increased theta and delta frequencies compared to a control group. It is clear from these behavioral and EEG findings that malnutrition at a young age has important acute deleterious effects on early brain function, cognition and behavior. However, the effects of early childhood malnutrition on brain function in adulthood are still unknown. Moreover, to our knowledge, no study has investigated brain function during a cognitive task.

The Barbados Nutrition Study (BNS) is a 55-year longitudinal study that has followed a Barbadian cohort hospitalized during the first year of life for a single episode of moderate to severe protein-energy malnutrition (PEM; Galler et al., 1983a,1987a) based on the Gomez classification (Gomez et al., 1955), and matched healthy controls who were former classmates of the PEM participants. The objectives of this study are to characterize the medical, neuropsychological, behavioral and brain effects of early PEM over the lifespan. The cohort comprises participants that were originally recruited between 1967 and 1972 when they were hospitalized as infants and subsequently enrolled in a government program that provided health and nutrition monitoring until the children reached 12 years of age. The malnutrition episode was restricted to the first year of life and all children in the PEM group achieved complete catch-up in physical growth by adolescence (Galler et al., 1987a,b,c). Control participants were classmates of the PEM children and were matched for age, sex, and handedness. Neuropsychological and psychiatric assessments revealed many cognitive, behavioral and mental health impairments associated with early childhood PEM, including lower IQ, conduct problems and higher prevalence of affective and depressive symptoms, as well as attention deficits (Galler et al., 1983a,b, 2010, 2012a). Additionally, most participants in the PEM group exhibit persistent attention and executive problems during childhood, adolescence and adulthood compared to the control group (Galler et al., 1983b,^1990^, 2012b; Waber et al., 2014a). In a recent study, brain function alterations were reported in the resting state activity of previously malnourished 5–11 years old children from the BNS cohort: specifically, an excess of theta, alpha2 and beta frequencies, and a decrease of alpha1, all suggesting a maturational lag in cortical development despite complete nutritional rehabilitation and demonstrated catch-up growth (Taboada-Crispi et al., 2018). However, little is known about the long-term effects of early childhood malnutrition on brain function in adults from this cohort.

Our aim was to study the persistence of brain function alterations in adults who experienced moderate to severe PEM during the first year of life by comparing the brain activity in adults from the PEM and the control groups of the BNS cohort using Evoked-Related Potentials (ERPs). Considering the attention and executive impairments previously reported in the PEM group, we administered a Go-No-Go attention task during EEG recordings to specifically isolate those altered processes. In a Go-No-Go task, two ERPs components are typically studied, namely the N2 and P3 components. N2 is defined as the largest fronto-central negative polarity deflection between 200 and 450 ms, and P3 is defined as the largest fronto-central positive polarity deflection between 350 and 600 ms (McLoughlin et al., 2010; Woltering et al., 2013; Grane et al., 2016; Schmüser et al., 2016). N2 and P3 are respectively, associated with conflict monitoring (i.e., conflict between the prepotent response and required response) and inhibition response (i.e., cancelation of a planned or prepotent response) processes in an attention/inhibition control task such as Go-No-Go, Stop signal, Stroop, or Flanker task (Braver et al., 2001; Smith et al., 2008). Those components have been widely studied and are thought to be altered in populations with attention and executive impairments like ADHD (McLoughlin et al., 2010; Johnstone et al., 2013; Woltering et al., 2013; Grane et al., 2016; Schmüser et al., 2016) and schizophrenia (Doege et al., 2010; Araki et al., 2016). In the current study, we hypothesized that the PEM group would show smaller N2 and P3 peak amplitudes absolute values compared to the healthy control group. This is the first study to investigate ERP adult brain function following early childhood malnutrition. It is also the first to examine the impact of childhood malnutrition on brain function during a cognitive task.

## Materials and Methods

### Site of Study

All study participants were born in Barbados, an English-speaking Caribbean country, between 1967 and 1972. In this era, the infant mortality rate in Barbados was 46 per 1,000 live births, compared with 7.8 per 1,000 live births today (UNICEF, 2021). Childhood malnutrition was common at this time and there were some indications that it was related to early weaning and choice of weaning food, but is now virtually eliminated because of an improved economy and an island-wide program to eradicate malnutrition (Galler et al., 1983b). Barbados has a population of 285,000 persons, primarily of African origin (92%), and the majority are lower middle class. The literacy rate is 99% and school attendance is obligatory to 17 years.

### Participants

Fifty-five adults, all born between 1967 and 1972, were recruited from the Barbados Nutrition Study (BNS; see Ramsey, 1979; Galler et al., 1983a,b for the first reports on this cohort) cohort during June–July 2018 and were approximately 45–51 years when tested. Briefly, the PEM group participants were hospitalized with moderate to severe PEM (Gomez et al., 1955) limited to the first year of life. Inclusion criteria were: (1) birth weight > 2,500 g (2) no pre- or postnatal complications (3) Apgar scores ≥ 8, (4) no encephalopathic events during childhood and (5) no further malnutrition after age one. The children were enrolled in an intervention program until 12 years of age, that provided subsidized foods, nutrition education, home visits, medical care and a preschool program two to three mornings a week (Ramsey, 1979). Healthy controls came from the same classrooms and met the same inclusion criteria but had no histories of malnutrition. They were matched to the PEM group by age, gender, and handedness (to allow comparison of neurodevelopmental measures). In the current study, two participants from the PEM group had to be excluded, one due to heavy alcohol consumption the night before testing, and the other because he could not complete the task due to discomfort. The final sample included 24 PEM participants and 29 controls.

This study has been performed in accordance with the ethical standards proposed in the 1964 Declaration of Helsinki and its later amendments. All study participants provided written informed consent and were compensated for their time and travel expenses. This study was approved by the Massachusetts General Hospital IRB (IRB Protocol 2015P000329/MGH), Hôpital Sainte-Justine, the Barbados Ministry of Health and Centro de Neurociencias de Cuba’s (2017/02/17/CNEURO) Ethics’ committees.

### Measures

A socioeconomic index measure was collected in the first BNS data collection when the participants were 5–11 years of age using the Ecology Questionnaire (Galler and Ramsey, 1985; Galler et al., 2012c) and was used in the current study to control for initial differences in childhood socioeconomic factors between malnourished and control groups. The questionnaire completed by parents/primary caretakers included items about conditions in the home and parental educational level and employment history. Principal component analysis using varimax rotation was computed on the questionnaire data and yielded a household standard of living factor, which was used in the current study as a childhood socioeconomic status (SES) measure (see Galler and Ramsey, 1985 for details and psychometric properties).

### Procedure

Electroencephalography (EEG) recordings took place at the Barbados Nutrition Study center, Bridgetown, Barbados. The room was air-conditioned and the temperature was maintained at 24°C. Participants were seated in a comfortable chair and fitted with a 21-EEG Ag/AgCl electrode active cap, one vertical and one horizontal electrooculogram (EOG), and one electrocardiogram (ECG). The EEG signal was recorded on the scalp using the actiCHamps amplifier and Brain Vision Recorder Software (Version 1.20, Brain Products, GMbH, Gilching, Germany). EEG channels were positioned according to the 10–20 system (Fz, Cz, Pz, Oz, Fp1/2, F3/4, C3/4, P3/4, O1/2, F7/8, T3/4, T5/6, plus ground-FPz and reference-FCz). Impedance was kept under 10 kΩ. A near-infrared spectroscopy (NIRS) recording was performed simultaneously with the EEG. However, the NIRS data are not included in the current article and will be reported in a subsequent publication. A Go-No-Go task was performed during the EEG-NIRS recording.

### Go-No-Go Task

The Go-No-Go task was presented using Presentation (version 20.2, Neurobehavioral Systems). Participants were instructed to click on a computer mouse as fast as possible using the index finger of their dominant hand whenever a letter appeared on the screen (Go trial), but to withhold from clicking if the letter was an X (No-Go trial). Each letter was presented pseudorandomly for 500 ms and disappeared as soon as a motor response was made. The inter-stimulus interval varied randomly between 700 and 1,000 ms. The task utilized a block design in which each block of 20 stimuli was interleaved with a resting period that varied randomly between 15 and 21 s. A block design was used rather than a single trial paradigm to accommodate both the EEG and NIRS signals. The task consisted of 14 No-Go blocks, and 14 Go blocks. The No-Go blocks included 30% of No-Go trials (6 X) and 70% of Go trials (14 letters). The Go blocks included Go trials only. Each participant went through a practice trial of one No-Go block that included feedback before the actual task. Overall, 28 blocks were presented, for a total of 476 Go and 84 No-Go trials (total of 560 letters). Additional blocks were presented so that at least 67 No-Go trials (80% of trials total number) were correctly inhibited to ensure a good signal-to-noise ratio. Three behavioral variables were computed based on the 28 blocks of the task (without additional blocks): reaction time (RT), No-Go accuracy and Go accuracy.

### Electroencephalography Recording and Data Processing

The EEG signal was recorded from the scalp using a 500 Hz sampling rate and referenced to electrode FCz. The data pre-processing was done using Brain Vision Analyzer (Brain Products, Munich, Germany). Data were first filtered offline between 0.5 and 35 Hz using a Butterworth filter with a notch filter at 50 Hz to remove any electrical interference. Ocular artifacts were then removed by subtracting the corresponding components using Independent Component Analysis (ICA). The data were then re-sampled at 512 Hz and re-referenced to the average reference. Each trial was segmented from −500 to 1,000 ms after stimulus onset. DC detrend was applied to the data to remove signal drift.

Artifact correction was then performed to reject any segment with artifacts for each channel individually. Any segment with a voltage step >50 μv/ms was removed. The allowed amplitude range was between 100 and −100 μv, with any value outside this range being rejected. A trained Ph.D. candidate (KR) then performed a visual inspection of the signal to detect any remaining artifacts and validate the artifact correction. Every incorrect trial, namely every No-Go followed by a motor response or Go without a motor response, was also rejected. A baseline correction was further applied using 200 ms before stimulus onset. Trials were then averaged in Correct No-Go [mean (*M*) = 69.6 trials, standard deviation (*SD*) = 10.5] and Correct Go trials (*M* = 471.3, *SD* = 67.3).

### Evoked-Related Potentials Analysis

**FIGURE 1:**
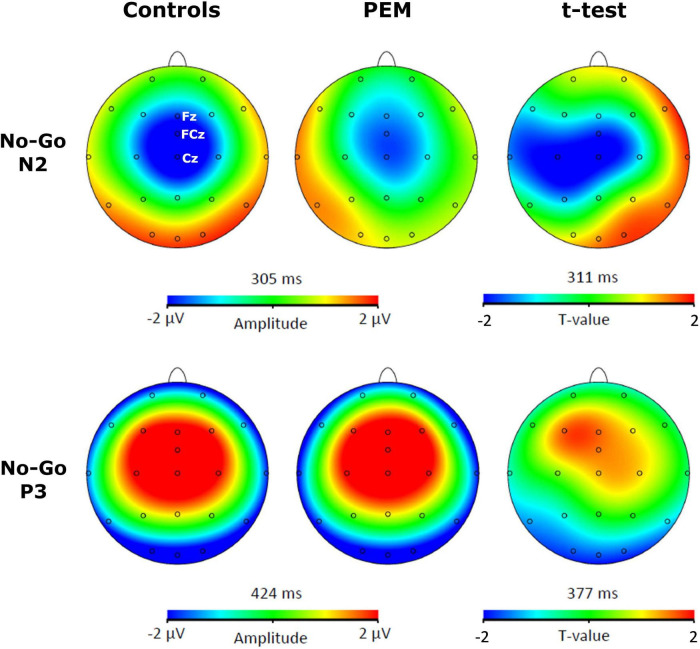
Topographic activation and topographic *t*-test (not corrected) of the difference of activation between protein-energy malnutrition (PEM) and control groups during No-Go condition [*t*(51) > 2.008, *p* < 0.05].

**FIGURE 2:**
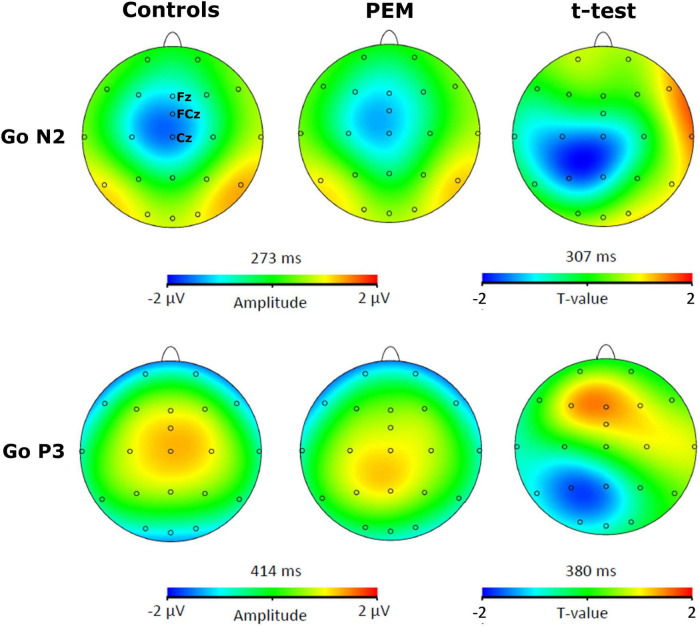
Topographic activation and topographic *t*-test (not corrected) of the difference of activation between PEM and control groups during Go condition [*t*(51) > 2.008, *p* < 0.05].

For each participant, a semi-automatic peak detection for the N2 and P3 ERP components was first performed on Correct No-Go and Correct Go trials individually on Fz, Cz, and FCz electrodes, where the components have previously been reported (Falkenstein et al., 1999; Donkers and van Boxtel, 2004). N2 was defined as the largest fronto-central negative polarity deflection between 200 and 450 ms and P3 was defined as the largest fronto-central positive polarity deflection between 350 and 600 ms (McLoughlin et al., 2010; Woltering et al., 2013; Grane et al., 2016; Schmüser et al., 2016). The peaks were reviewed by a trained Ph.D. student (KR) and an expert in EEG processing (PV). The absolute value of the mean amplitude ±10 ms around the peak and the latency of each peak were extracted and used for statistical analyses. For example, for a negative component such as N2 for a given participant, if the peak was detected at 300 ms, we took the absolute value of the mean amplitude from 290 to 310 ms (considering our sampling rate is at 512 Hz, this means averaging five sample points before and after the peak). Thus, the term amplitude used in the current manuscript refers to the absolute value of the mean amplitude around the peak. Brain Vision assisted topographic *t*-tests (without correction for multiple comparisons) were performed to compare amplitude topographies of N2 and P3 components between Nutrition groups (PEM vs. Control) for No-Go and Go conditions individually when group differences were maximal (see Figures 1, 2).

### Statistical Analyses

Data were analyzed using SPSS statistical software, version 25. For the behavioral measures group differences in reaction time, Go accuracy and No-Go accuracy on the Go-No-Go task were analyzed using three separate independent sample *t*-tests with Nutrition Group as the between-subjects factor. For the ERP components, group differences were tested using four mixed ANCOVAs [3 (Electrode: Fz, Cz, FCz) × 2 (Condition: No-Go, Go) × 2 (Nutrition Group: Controls, PEM)] performed separately for N2 and P3 and for their amplitude and latency. The childhood SES factor was included as a covariate in all analyses. The significant p-value was set to *p* ≤ 0.05. The extreme outliers, defined as a value that is over three times the interquartile range, were winsorized, as suggested by Wilcox (2012). Greenhouse-Geisser adjustment was applied to account for any departure of sphericity (the less conservative Huynh-Feldt correction provided similar results) and the Bonferroni correction was applied for multiple comparisons. A Mann-Whitney *U*-test was used if the Shapiro-Wilk normality test was violated. When non-parametric and parametric tests provided similar results, parametric tests were reported.

## Results

### Demographic Characteristics

The demographic characteristics of the sample are reported in Table 1. The two nutrition groups did not differ in terms of age, gender or handedness, but there were significant differences in childhood standard of living indicating lower SES (childhood ecology factor scores) in the PEM vs. Control group.

**TABLE 1:** Demographic characteristics of participants.

	PEM	Control	t/χ^2^	*P*
**Demographic overview**				
*n*	24	29	0.47	0.49
Males [*N* (%)]	13 (54%)	14 (48%)	0.67	0.78
Age (years)	48.61 ± 1.87	48.65 ± 1.96	0.08	0.94
Handedness [*N* left (%)]	3 (13%)	3 (10%)	0.06	0.75
Childhood ecology factor (time 1)	−1.18 ± 0.75	−0.27 ± 0.77	4.32	<0.0001
Hollingshead educational score	5.13 ± 0.34	3.79 ± 1.54	4.52	<0.0001
Hollingshead occupational score	6.25 ± 1.03	4.31 ± 1.58	5.36	<0.0001
Income ($BDS)	325.10 ± 245.40	751.00 ± 717.60	2.99	<0.0001
**Medical history**				
Alcoholism	7 (29%)	6 (21%)	0.51	0.48
Cannabis abuse	3 (12%)	0 (0%)	N/A	
Diabetes	4 (17%)	2 (7%)	1.90	0.39
Hypertension	14 (58%)	17 (59%)	0.00	0.98
Encephalopathic events	7 (29%)	8 (28%)	0.02	0.90

*Handedness and Diabetes statistical tests were done using Fisher Exact Test (non-parametric). The Hollingshead scores were measured using the Hollingshead scales (Hollingshead, 1957), which rank social position based on occupation and educational attainment.*

### Original Cohort Comparison

Analysis to determine the representativeness of the current sample relative to the original Wave 1 cohort yielded no significant differences between current study participants (*N* = 53) and non-participants (*N* = 205) who were studied in earlier waves with respect to gender (percent male: χ^2^ = 1.93, *p* = 0.17), history of childhood malnutrition (χ^2^ = 0.59, *p* = 0.44), childhood ecology (*t* = 1.11, *p* = 0.27; see Galler et al., 1983b for Wave 1 details). The results are presented in Supplementary Table 1.

### Behavioral Results

Table 2 displays the means and standard deviations of the behavioral measures. The nutrition groups differed on Go accuracy (*U* = 462, *p* = 0.041), with greater Go accuracy for the control group compared to the PEM group. However, there were no group differences in reaction time or No-Go accuracy.

**TABLE 2:** Means and standard deviations of behavioral measures for protein-energy malnutrition (PEM) and control groups.

Measure		PEM (*n* = 24)				Control (*n* = 29)				Mann-Whitney *U*		*P*
								
		M		SD		M		SD		
Reaction time (ms)		357.95		35.24		357.83		38.30		352.00		0.94
Go accuracy (%)		97.09		3.22		98.25		2.59		462.00		0.04
No-Go accuracy (%)		76.64		12.11		80.21		12.87		420.00		0.20

### Evoked-Related Potentials Results: N2

**FIGURE 3:**
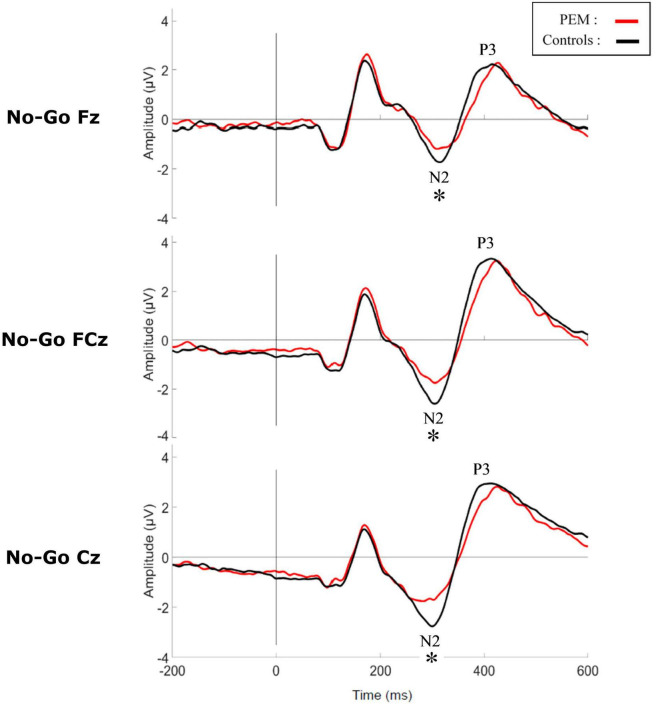
Grand average waveform of the N2 and P3 components during No-Go condition for each group. *p < 0.05.

**FIGURE 4:**
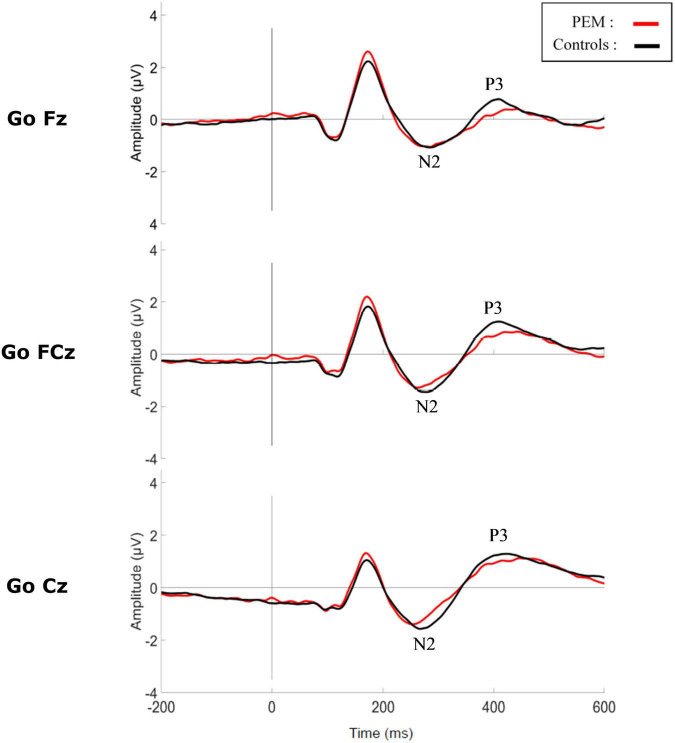
Grand average waveform of the N2 and P3 components during Go condition for each group.

Figures 3, 4 shows the waveform of the N2 and P3 components for each nutrition group during the No-Go and Go conditions, respectively. Either controlling or not for the SES, results revealed no differences regarding Nutrition Group effects. Nevertheless, since there was a difference in the SES between groups, we report here the results while controlling for the SES.

#### Amplitude

The three-way ANCOVA revealed a main effect of Condition [*F* (1,50) = 12.928, *p* = 0.001, η_p_^2^ = 0.205] with the No-Go condition generating a larger N2 amplitude than the Go condition, according to pairwise comparisons. Significant interactions between Condition and Nutrition Group [*F* (1,50) = 4.877, *p* = 0.032, η_p_^2^ = 0.089] and between Electrode and Nutrition Group ([*F* (2,100) = 3.693, *p* = 0.051, η_p_^2^ = 0.069] were found. Appropriate one-way ANCOVAs were conducted to further explore these interactions.

For the Condition x Nutrition Group interaction, one-way ANCOVA revealed a significant difference in the N2 amplitude between the two groups but only in the No-Go condition [No-Go: *F* (1,50) = 4.025, *p* = 0. 050, η_p_^2^ = 0.075; Go: *F* (1,50) = 0.112, *p* = 0.740, η_p_^2^ = 0.002]. During this condition, the amplitude of N2 was significantly larger for the control group compared to the PEM group.

For the Electrode × Nutrition Group interaction, the one-way ANCOVA revealed a smaller amplitude of the N2 in the PEM group compared to the control group, but only for Cz [Cz: *F* (1,50) = 4.327, *p* = 0.043, η_p_^2^ = 0.080; Fz: *F* (1,50) = 0.096, *p* = 0.758, η_p_^2^ = 0.002; FCz: *F* (1,50) = 2.236, *p* = 0.141, η_p_^2^ = 0.043].

#### Latency

The three-way ANCOVA showed a main effect of Electrodes [*F* (2,100) = 9.239, *p* < 0.001, η_p_^2^ = 0.156] with pairwise comparisons indicating a shorter latency for Cz compared to FCz (*p* < 0.001) and Fz (*p* = 0.001). There was also a main effect of Condition [*F* (1,50) = 9.784, *p* = 0.003, η_p_^2^ = 0.164] with the No-Go condition generating a longer N2 latency than the Go condition. No interaction was significant, and there was no significant nutrition group effect.

### Evoked-Related Potentials Results: P3

#### Amplitude

The three-way ANCOVA revealed a main effect of Electrodes [*F* (2,100) = 11.528, *p* < 0.001, η_p_^2^ = 0.187] with pairwise comparisons indicating a smaller amplitude for Fz compared to FCz (*p* < 0.001) and Cz (*p* = 0.001), and for FCz compared to Cz (*p* = 0.056). There was also a main effect of Condition (*F* (1,50) = 225.173, *p* < 0.001, η_p_^2^ = 0.690) with the No-Go condition generating a larger P3 amplitude than the Go condition, according to pairwise comparisons. There was also a significant interaction between Electrode and Condition [*F* (2,100) = 4.299, *p* = 0.034, η_p_^2^ = 0.079]. A repeated measures ANCOVA was conducted as a *post hoc* analysis to further explore this interaction. For the No-Go condition [*F* (2,102) = 11.916, *p* < 0.001, η_p_^2^ = 0.189], P3 amplitude was significantly larger at FCz compared to Fz (*p* < 0.001) and Cz (*p* = 0.013), and was larger at Cz compared to Fz (*p* = 0.028). For the Go condition [*F* (2,102) = 14.731, *p* < 0.001, η_p_^2^ = 0.224], P3 amplitude was larger at FCz compared to Fz (*p* < 0.001) and larger at Cz compared to Fz (*p* < 0.001). There was no significant effect of the Nutrition Group on the measure.

#### Latency

The three-way ANCOVA revealed a main effect of Condition [*F* (1,50) = 4.885, *p* = 0.032, η_p_^2^ = 0.089] with the No-Go condition generating a longer P3 latency than the Go condition, according to pairwise comparisons. Significant interactions between Electrode and Nutrition Group [*F* (2,100) = 3.339, *p* = 0.045, η_p_^2^ = 0.063] and between Condition and SES [*F* (1,50) = 4.989, *p* = 0.03, η_p_^2^ = 0.091] were found. Appropriate one-way ANCOVAs and correlations were conducted to further explore these interactions.

For the Electrode × Nutrition Group interaction, the one-way ANCOVA revealed no significant difference in P3 latency between groups for any electrode [Fz: *F* (1,50) = 0.023, *p* = 0.879, η_p_^2^ < 0.001; FCz: *F* (1,50) = 0.212, *p* = 0.647, η_p_^2^ = 0.004; Cz: *F* (1,50) = 0.174, *p* = 0.678, η_p_^2^ = 0.003]. There was also no significant difference in P3 latency between electrodes for any group [PEM: *F* (2,44) = 2.499, *p* = 0.106, η_p_^2^ = 0.102; Controls: *F* (2,54) = 0.734, *p* = 0.448, η_p_^2^ = 0.026].

For the Condition × SES interaction, the correlations revealed no significant relationships between P3 latency and SES for the Go condition (*r* = 0.035, *p* = 0.804) or the No-Go condition (*r* = −0.193, *p* = 0.167).

Tables 3–5 display means and standard deviations of the ERP measures for each condition for Fz, Cz, and FCz, respectively.

**TABLE 3:** Mean and standard deviation of the evoked-related potentials (ERP) measures for Fz electrode.

Measure	PEM (*n* = 24)		Control (*n* = 29)	
	M	SD	M	SD
No-Go amplitude N2	−1.84	1.44	−2.20	1.38
Go amplitude N2	−1.48	0.92	−1.36	1.04
No-Go amplitude P3	2.72	2.21	3.02	1.57
Go amplitude P3	0.83	1.06	0.98	0.88
No-Go latency N2	313.88	36.03	313.24	35.65
Go latency N2	291.75	36.4	294.85	35.2
No-Go latency P3	417.24	26.3	415.75	40.1
Go latency P3	416.34	36.34	414.8	39.23

**TABLE 4:** Mean and standard deviation of the ERP measures for FCz electrode.

Measure	PEM (*n* = 24)		Control (*n* = 29)	
	M	SD	M	SD
No-Go amplitude N2	−2.04	1.78	−3.07	1.53
Go amplitude N2	−1.53	0.97	−1.72	1.23
No-Go amplitude P3	3.38	1.52	3.70	1.80
Go amplitude P3	1.33	0.97	1.53	0.97
No-Go latency N2	305.09	37.43	301.19	30.39
Go latency N2	279.70	41.46	281.92	36.56
No-Go latency P3	417.48	24.47	420.39	44.71
Go latency P3	416.10	37.53	413.46	40.61

**TABLE 5:** Mean and standard deviation of the ERP measures for Cz electrode.

Measure	PEM (*n* = 24)		Control (*n* = 29)	
	M	SD	M	SD
No-Go amplitude N2	−2.19	1.63	−3.06	1.52
Go amplitude N2	−1.61	1.02	−1.66	1.23
No-Go amplitude P3	3.74	2.19	4.24	1.76
Go amplitude P3	1.21	0.91	1.55	1.02
No-Go latency N2	307.94	36.42	310.01	23.93
Go latency N2	287.60	37.51	291.49	33.66
No-Go latency P3	426.76	34.67	416.15	39.51
Go latency P3	415.93	37.06	412.85	38.25

## Discussion

The main objective of this study was to compare the brain activity of adults who experienced moderate to severe PEM during the first year of life and healthy controls with no histories of malnutrition using a Go-No-Go inhibition task. We hypothesized that the PEM group would show altered neural response associated with attention and inhibition (N2 and P3 components) during the task. The hypothesis was partially confirmed, since we observed a reduction of N2 amplitude during the No-Go condition in the PEM group compared to the control group, but there was no difference detected in P3. Overall, results of the Go-No-Go task revealed a main effect of Condition for both components (N2 and P3) in both PEM and control groups. This is a typical result for a Go-No-Go task, indicating that the No-Go condition induces a genuine inhibition response which delays the onset and amplifies the N2 and P3 components (Eimer, 1993; Bokura et al., 2001; Schmüser et al., 2016).

The N2 amplitude was smaller in the PEM group compared to the control group during the No-Go condition. This result has also been found in adults and children with ADHD (Brandeis et al., 2002; Fallgatter et al., 2004; Gow et al., 2012; Woltering et al., 2013). Of note, attentional deficits have also been reported in the PEM participants in earlier studies (Galler et al., 1983a,^1990^, 2012b; Waber et al., 2014a). There is debate amongst the scientific community on the cognitive process underlying the N2 component. Although some authors argue that N2 is related to the inhibition process (i.e., cancelation of a planned or prepotent response; Eimer, 1993; Falkenstein et al., 1999), more recent studies point to an association between N2 and conflict monitoring (i.e., conflict between the prepotent response and required response; Donkers and van Boxtel, 2004; Enriquez-Geppert et al., 2010; Huster et al., 2013; Groom and Cragg, 2015). According to the conflict monitoring hypothesis, an inhibition task such as Go-No-Go should evoke an N2 component because of the unbalanced ratio between Go and No-Go trials, which would lead to the creation of a prepotent response (Go) that conflicts with the infrequent inhibition of this response (No-Go) and not because of the inhibition process *per se* (Braver et al., 2001). However, conflict monitoring is closely related to attention, since it is responsible for triggering cognitive control changes by adjusting attention levels to optimize performance and prevent subsequent conflict (Botvinick et al., 2001). According to these models, our results can be interpreted as showing impairment in conflict monitoring and/or attention following early childhood malnutrition.

Surprisingly, the ERP results showed no difference between the two nutrition groups in P3 amplitude or latency. P3 component is usually considered to be a marker of response inhibition processing and evaluation (Huster et al., 2013; Groom and Cragg, 2015). In studies of ADHD using a Go-No-Go task, both components, N2 and P3 are typically altered (Johnstone et al., 2013; Woltering et al., 2013). However, the reduced N2 amplitude in the PEM group and similar P3 amplitude in the PEM and control groups is in line with the behavioral results. Indeed, in the current study, Go accuracy was significantly lower in the PEM group compared to the control group, revealing that adults who suffered from early childhood malnutrition committed more omission errors than controls. No difference in commission error rate was found. In this respect, it was previously reported in this cohort that inattention symptoms were more prevalent in the PEM group than impulsivity, and that omission errors differentiated the nutrition groups better than commission errors on the Connors Continuous Performance Task-II (Galler et al., 2012b). Omission errors are usually attributed to impairments in attention and vigilance whereas commission errors are associated with inhibition deficits. Therefore, early childhood malnutrition may be more closely associated with diminished attention and vigilance. Furthermore, no differences in reaction times were found between our groups. Slower reaction times have been consistently reported in populations with attentional and inhibition difficulties and are associated with hyperactivity and impulsivity (Wiersema et al., 2006; Rubia et al., 2007; Barkley et al., 2008; McLoughlin et al., 2010; Tamm et al., 2012; Schmüser et al., 2016).

The behavioral results are in line with the persistent attention deficits previously reported in our cohort during childhood, adolescence and adulthood (Galler et al., 1983a,^1990^, 2012b; Waber et al., 2014a). Inattention has also been reported in other studies assessing the neurocognitive profile of malnourished populations (Richardson et al., 1972) and following famine exposure in early childhood (Wang et al., 2016).

The altered electrophysiological marker found in this study is also coherent with the literature on brain function effects following childhood malnutrition. Indeed, several studies show that childhood malnutrition is associated with the slowing of the EEG’s dominant rhythm in infancy (Stoch et al., 1963; Baraitser and Evans, 1969; Griesel et al., 1990; Gladstone et al., 2014) and electrophysiological alterations that persist in childhood even after nutritional rehabilitation (Bartel et al., 1979; Taboada-Crispi et al., 2018). Recently, our colleagues also found abnormal developmental resting-state EEG source activity when comparing childhood and adulthood EEGs in the BNS cohort, with PEM group showing an increase in low alpha, a decrease in high alpha and no changes in beta activity whereas control group showed a slight decrease in low alpha, an increase in high alpha and a decrease in beta activity (Bosch-Bayard et al., 2022). More specifically in ERP studies, ERP abnormalities were also found in concurrently malnourished infants with a higher relative abnormality of their auditory evoked potentials compared to controls (Barnet et al., 1978; McDonald et al., 2007). Long-lasting alterations in ERP components during a recognition memory task were also found in children 10 years after iron deficiency treatment (Congdon et al., 2012). We extend knowledge in this field by showing brain function deficits still perceptible in adulthood.

Experimental studies further confirm the findings of the current study. Prenatally malnourished rats also show a significant impairment in executive function and attentional tasks (McGaughy et al., 2014; Newman et al., 2019). Further, using 2-deoxyglucose to measure neural activity during an attentional task, it has been shown that malnourished and control rats recruit different brain pathways (Rushmore et al., 2021). Whereas prefrontal activity was a predictor of task performance in controls, malnourished rats relied on brain networks involving limbic structures such as the hippocampal formation, reflecting compensatory brain responses to malnutrition. As in the current study, animal research has also shown alterations in brain function when performing attentional and executive tasks.

The analyses presented in this study adjusted for childhood socioeconomic and home environmental conditions of the participants. Indeed, given the known impact of the SES on brain function (Farah, 2017; Yaple and Yu, 2020), it was important to control for the initial childhood socioeconomic differences between our two groups. Although SES effects did not modify the significant effects of early malnutrition on executive function in the BNS cohort (Galler et al., 2012b; Waber et al., 2014a), lower SES has been associated with impaired performance in executive functions and attention shifting tasks (Lawson et al., 2018) as well as differences in ERP components during attentional tasks (St. John et al., 2019; Hiran Perera et al., 2021). Thus, by controlling for the SES, the presented results suggest that malnutrition limited to the first year of life is associated with abnormalities in adult brain function that go beyond what could be attributable to socioeconomic factors.

This study has several limitations. First, the small sample size does not allow us to conclude with certainty that the results were not due to a lack of statistical power. Nevertheless, the effect size is moderate for the N2 amplitude difference between the groups (*d* = 0.55). Additionally, the results reported here are specific to the N2 and P3 electrophysiological markers and different analyses might reveal other electrophysiological differences between our groups. For example, the use of source localization analysis on our data would allow for a more precise exploration at the source level of the temporal dynamics of brain functioning in executive function. Also, spectral analysis or functional connectivity analysis could reveal interesting information on the brain functional organization. Correlations between physiological data and EEG data could be relevant as well, for instance to study the relationship between heart rate variability and cognitive decline (Forte et al., 2019). Further analyses will be performed in a near future to better characterize and compare brain functions in the two groups. We also plan on comparing our task-based EEG outcomes with the resting-state EEG outcomes found in this cohort by our collaborators (Taboada-Crispi et al., 2018; Bosch-Bayard et al., 2022).

## Conclusion

In sum, this study shows that adults who experienced early childhood malnutrition limited to the first year of life have different brain response patterns during a response inhibition task compared to healthy controls. This adds to the existing literature on cognitive and neural outcomes following early childhood malnutrition, suggesting that attention and conflict monitoring, two cognitive control processes, remain altered in adulthood. Considering the impact of persisting cognitive alterations on the quality of life, more research is needed to better characterize early brain markers and clinical profiles associated with early childhood malnutrition in order to develop a disease progression model applicable to vulnerable populations.

## Data Availability Statement

The data that supports the findings of this study is available from the corresponding author AG, anne.gallagher@umontreal.ca upon reasonable request.

## Ethics Statement

The studies involving human participants were reviewed and approved by Massachusetts General Hospital’s IRB, Sainte-Justine University Hospital’s Ethic Committee, Centro de Neurociencias de Cuba’s Ethic Committee, and the Barbados Ministry of Health. The patients/participants provided their written informed consent to participate in this study.

## Author Contributions

KR acquired the data, performed the analysis, wrote the original draft, and revised the manuscript. PV participated in data acquisition and data analysis and revised the manuscript. JT provided help in data analysis and revised the manuscript. MB participated in the conceptualization of the study and revised the manuscript. CB coordinated the data acquisition. AR helped the coordination of the data acquisition and revised the manuscript. PV-S and AG conceptualized the study, provided supervision, and revised the manuscript. JG conceptualized the study, coordinated the data acquisition, provided supervision, and revised the manuscript. All authors contributed to the article and approved the submitted version.

## Conflict of Interest

The authors declare that the research was conducted in the absence of any commercial or financial relationships that could be construed as a potential conflict of interest.

## Publisher’s Note

All claims expressed in this article are solely those of the authors and do not necessarily represent those of their affiliated organizations, or those of the publisher, the editors and the reviewers. Any product that may be evaluated in this article, or claim that may be made by its manufacturer, is not guaranteed or endorsed by the publisher.
