# Supplementary material for: Impact of Early Childhood Malnutrition on Adult Brain Function: An Evoked-Related Potentials Study
Source: Front Hum Neurosci. 2022 Jul 1;16:884251. doi: 10.3389/fnhum.2022.884251 (PMC9283562; doi:10.3389/fnhum.2022.884251)
Supplement: Supplementary file 1 [file Table_1.DOCX]

Supplementary Material

# Supplementary Figures and Tables

| Supplementary Table 1. *Demographic Characteristics of Participants vs. Non-Participants from Original Cohort* | | | | |
| --- | --- | --- | --- | --- |
| Characteristic | Participant | Non-participant | t-test / χ2 | *p* |
| *N* | 53 | 205 |  |  |
| Male [*N* (%)] | 27 (50.90) | 126 (61.50) | 1.93 | 0.17 |
| History of Malnutrition [*N* (%)] | 24 (45.30) | 105 (51.20) | 0.59 | 0.44 |
| Childhood Ecology (*SD*) | -0.68 (0.88) | -0.53 (1.00) | 1.11 | 0.27 |
